# Food Group Intakes as Determinants of Iodine Status among US Adult Population

**DOI:** 10.3390/nu8060325

**Published:** 2016-05-26

**Authors:** Kyung Won Lee, Dayeon Shin, Mi Sook Cho, Won O. Song

**Affiliations:** 1 Department of Food Science and Human Nutrition, Michigan State University, 469 Wilson Road, Trout FSHN Building, East Lansing, MI 48824, USA; kyungwon@msu.edu (K.W.L.); shinda@msu.edu (D.S.); 2 Department of Nutritional Science and Food Management, Ewha Womans University, Human Ecology Building, 52, Ewhayeodae-gil, Sodaemun-gu, Seoul 03760, Korea; misocho@ewha.ac.kr

**Keywords:** iodine status, urinary iodine concentration, food group intake, National Health and Nutrition Examination Survey

## Abstract

Adequate intake of iodine is essential for proper thyroid function. Although dietary reference intakes for iodine have been established, iodine intake cannot be estimated due to the lack of data on iodine contents in foods. We aimed to determine if food group intakes can predict iodine status assessed by urinary iodine concentration (UIC) from spot urine samples of 5967 US adults in the National Health and Nutrition Examination Survey (NHANES) 2007–2012. From an in-person 24-h dietary recall, all foods consumed were aggregated into 12 main food groups using the individual food code of the US Department of Agriculture (USDA); dairy products, meat/poultry, fish/seaweed, eggs, legumes/nuts/seeds, breads, other grain products, fruits, vegetables, fats/oils, sugars/sweets, and beverages. Chi-square test, Spearman correlation, and multiple linear regression analyses were conducted to investigate the predictability of food group intakes in iodine status assessed by UIC. From the multiple linear regressions, the consumption of dairy products, eggs, and breads, and iodine-containing supplement use were positively associated with UIC, whereas beverage consumption was negatively associated with UIC. Among various food group intakes, dairy product intake was the most important determinant of iodine status in both US men and women. Subpopulation groups with a high risk of iodine deficiency may need nutritional education regarding the consumption of dairy products, eggs, and breads to maintain an adequate iodine status. Efforts toward a better understanding of iodine content in each food and a continued monitoring of iodine status within US adults are both warranted.

## 1. Introduction

Iodine is a trace element, of which only a small amount is needed in the body. Adequate intake of iodine is indispensable to the synthesis of thyroid hormones, thyroxine (T4), and tri-iodothyronine (T3), which are required for growth, organ development, and utilization of nutrients by the body [1]. Severe iodine deficiency increases the risk of stillbirths and neonatal death and causes cretinism, developmental failure, delayed cognition development in childhood [2], mental retardation, hypothyroidism, and goiter in adults [3]. In addition, iodine deficiency is regarded as a leading preventable cause of mental retardation and one of the most serious global health issues [4]. Health risks have been documented not only for severe iodine deficiencies, but also as consequences of mild to moderate iodine deficiencies that are present in the US [5] and other developed countries [6]. Mild to moderate iodine deficiencies are associated with lower mental and cognitive function resulting in low IQ in children and decreased work productivity, metabolic abnormalities, thyroid dysfunction, and even goiter in adults [7,8,9,10].

Serious efforts have been undertaken, globally, to eradicate iodine deficiency. In 1993, the World Health Organization (WHO), the United Nations Children’s Fund, and the Iodine Global Network (formerly International Council for Control of Iodine Deficiency Disorders) introduced universal salt iodization as a safe and cost-effective strategy to combat iodine deficiency. Salt had high feasibility and sustainability of fortification because it was consumed in almost all countries. They used 30–100 parts per million potassium iodate for salt fortification. This strategy was to guarantee optimal iodine intake for all individuals [11]. The organizations also recommended taking iodine-containing supplements for those living in areas with insufficient iodine but without salt iodization programs or that were vulnerable to iodine deficiency [12]. Continuous monitoring of iodine status and salt iodization programs, and continued interest of political support to prevent iodine deficiency, have also been suggested.

Despite such efforts, iodine deficiency has become a re-emerging public health concern in such industrialized countries as Australia and the United Kingdom (UK), which were once was regarded as iodine-sufficient countries [13,14,15,16]. In contrast to developing countries and the selected industrialized countries with re-emerging iodine deficiencies, the US has not yet been reported to have iodine deficiency as a serious public health issue [17]. However, researchers have reported a downward trend of median urinary iodine concentrations (UICs) in the US. Others reported that such subpopulation groups as pregnant or reproductive-aged women are at risk for mild iodine deficiency based on UIC [18,19]. In order to prevent iodine deficiency becoming a public health problem in the US, dietary sources of iodine need to be identified with continuous monitoring of iodine status measured by dietary intakes and biomarkers such as UIC, a valid, economical, and reliable method [20,21,22,23].

Although adequate dietary iodine intake is important in human development, only a few studies reported estimates of dietary exposure to iodine in the US and the world [22,23,24]. In general terms, the leading sources of dietary iodine are dairy products, eggs, fish, and seaweeds [22,25]. To help people estimate the iodine contents in total daily diet [26], the US Department of Agriculture (USDA) proposed a percentage daily value (% DV) set at 150 µg (100% DV) for iodine among US adults. For example, one cup of reduced fat milk and low fat plain yogurt provide 56 µg (37% DV) and 75 µg (50% DV), respectively. A large egg gives 24 µg (16% DV). Three ounces of fish sticks and baked cod may contribute 54 µg (36% DV) and 99 µg (66% DV) of iodine, respectively. One gram of dried or powdered seaweed, such as nori, wakame, kelp, or kombu, contains iodine in large ranges from 16 µg (11% DV) to 2984 µg (1989% DV) [27]. Another major dietary source of iodine is iodized table salt [28], which was introduced in the 1920s and proven to be effective in combating iodine deficiency in the US. Many salts sold in the US and in the world follow the current flexible recommendations for iodine fortification levels as a form of potassium iodate and potassium iodide [29]. The Salt Institute has estimated that about 70% of the table salt in the US is iodized, whereas table salt accounts for only 15% of total daily intake of salt [30,31]. To make matters worse in the US, processed foods or restaurant foods tend to use non-iodized salt due to the alleged adverse effect on the quality and taste of food [32,33]. Although iodized table salt is still an important source of iodine in the US [28], adequate iodine intake may not be fully achieved with iodized salt only. Thus, it is important to identify dietary sources that are compatible with recommended dietary allowances to sustain adequate iodine status [34].

Generally, dietary exposure to practically all known nutrients and biologically-active non-nutritive compounds can be estimated based on the USDA food-composition tables [35]. However, despite the Institute of Medicine’s dietary reference intake for iodine, the USDA food-composition tables do not include iodine contents, presumably due to high variability of iodine in the same kinds of foods [36]. Consequently, one cannot estimate dietary iodine intake with food-composition tables. Previous studies in which food group intakes were associated with UIC focused only on key dietary sources of iodine such as dairy products, grains, and dietary supplements [28,37,38]. In our study, we attempted to examine comprehensive food groups as a determinant of iodine status assessed by UIC, a valid biomarker to assess iodine status of a group. Therefore, we specifically tested the hypothesis that food group intake can be a determinant of iodine status in a subpopulation in the US using a food group-based approach rather than a single food item-based approach. We aimed to investigate UIC and prevalence of <50 µg/L in relation to food group intakes among the US adult population. In a situation that dietary iodine intake from single food items cannot be estimated by the USDA food-composition tables, a food group-based approach used in this study could provide a better possibility of the association between dietary intakes and iodine status in US populations.

## 2. Materials and Methods

### 2.1. Data Source and Study Population

The National Health and Nutrition Examination Survey (NHANES) has been conducted by the Centers for Disease Control and Prevention (CDC). The NHANES that began in the early 1960s has been conducted continuously in two-year cycles since 1999 to monitor changes in health and nutritional issues at the national level. Each survey of the NHANES collects cross-sectional data continuously from an annual stratified, multistage, and national probability sample of approximately 5000 civilian, non-institutionalized individuals in the US [39,40]. The NHANES survey collects a variety of information, including sociodemographic, dietary intake, health-related questions, and laboratory tests administered by trained medical technicians [41].

For this study, we merged the data collected through three cycles: 2007–2008, 2009–2010, and 2011–2012. We mainly focused on US adult population in this study because there has been less concern about iodine deficiency in US children and adolescents [38,42]. A total of 6679 participants aged 20 years and older and having UIC data were included in the analytic sample. Excluded were those who were pregnant and lactating women (*n* = 101), had energy intakes of less than 500 kcal or greater than 5000 kcal (*n* = 552), and had incomplete datasets on any of the sociodemographic and iodine intake-related variables (*n* = 59). Finally, 5967 US adults with UIC, 24-h recalls, and sociodemographic information were included in this study.

### 2.2. Iodine Status

About 90% of daily dietary iodine intake is excreted in the urine and measured values indicate a relatively steady state. UIC obtained from a spot urine sample is widely used as an indicator for assessing recent iodine intake of populations [2,43]. Spot urine samples have been collected in all NHANES except for NHANS II (1976–1980) from representative US residents aged six years and older at mobile examination centers. UIC was analyzed by this method by Caldwell *et al.* [19,44]. UICs of the six-year NHANES were measured by the same equipment and lab methods in the same lab site [45,46]. The WHO recommends that iodine status of a population be assessed by the median UIC of spot urine sample from representative large populations [41]. We used WHO recommendations for evaluating iodine status. In normal healthy adults, excluding pregnant and lactating women, iodine status is currently defined by the WHO as deficient (median UIC <100 µg/L), adequate (median UIC, 100–199 µg/L), more than adequate (median UIC, 200–299 µg/L), and excess (median UIC ≥300 µg/L). Additionally, the WHO provides guidelines specifying that the proportion of the total healthy population with UIC <50 µg/L should be with no more than 20% of it [41]. Therefore, we assessed the iodine status using median UICs at the population level and examined the prevalence of instances where <50 µg/L UIC. Each sample collected is weighted to adjust for oversampling and to generate representative results for the US adult population.

### 2.3. Dietary Intake and Classification of Food Group

The NHANES has two 24-h dietary recall information obtained by trained interviewers. One is an in-person 24-h dietary recall and the other is a subsequent 24-h dietary recall done by telephone. The USDA’s Automated Multiple-Pass Method was used to collect both in-person and phone 24-h dietary recall information [47]. Every food and beverage consumed by every NHANES participant has a unique eight-digit USDA food code, which is recorded on the 24-h dietary recall dataset. To understand the association between UIC and dietary intake data, we used only the first day’s single 24-h dietary recall collected from each participant obtained simultaneously with the urine sample. Since the first digit of the USDA food code makes it possible to recognize nine major food groups (milk/milk products; meat/poultry/fish; eggs; legumes/nuts/seeds; grain products; fruits; vegetables; fats/oils; sugars/sweets/beverages), we can understand and systematize, hierarchically, each participant’s mass information on dietary intake. However, the USDA food group is not subdivided enough to achieve the purpose of a study investigating the primary food sources of dietary iodine. Previous literature has reported that dairy products [22,38], fish/seaweed [25,37], and breads [23] are good sources of dietary iodine. We converted nine USDA food groups into 12 new food groups (meat/poultry; eggs; legumes/nuts/seeds; other grain products; fruits; vegetables; sugars/sweets; fats/oils; beverages), including food groups known to be high in iodine content (dairy products; fish/seaweed; breads).

### 2.4. Covariates

The study subjects were sub-grouped by sociodemographic and behavioral variables; sex (men and women); age (20–39, 40–59, ≥60 years of age); and race/ethnicity (non-Hispanic whites, non-Hispanic blacks, all Hispanics, other). Behavioral factors, including salt use at the table and in preparation, and iodine-containing supplement use, were self-reported. Table salt use was estimated by response to the question “How often do you add salt to your food at the table?” Responses were categorized into two categories, rarely and occasionally/very often. Information on salt use during food preparation was collected by asking “How often is ordinary salt or seasoned salt added in cooking or preparing foods in your household?” Salt use in preparation was categorized into two categories, never/rarely and occasionally/very often. Iodine-containing supplement use information was collected as a part of the NHANES dietary supplement questionnaire for all participants. Survey participants were asked to respond to the questionnaire that examines dietary supplement use in the previous 24 h. If a participant reported use of any dietary supplements, the interviewer from the National Center for Health Statistics would ask them to provide the container and/or label of the products with detailed information on the product name, and frequency and quantity of the use [48]. For the specific iodine-containing supplement use analysis, we identified supplements by their inclusion of iodine based on their ingredient identification codes on the dietary supplement database file. After matching the consumed dietary supplement product with the ingredient information from the database, we categorized participants as user or nonuser of dietary supplements with iodine in the previous 24 h.

### 2.5. Statistical Analyses

All statistical analyses in this study were performed using SAS (version 9.3, SAS Institute Inc., Cary, NC, USA). The NHANES is based on a multistage sampling design considering oversampling, stratification, and clustering, rather than simple random sampling data. To account for the complex survey design of the NHANES data, SURVEY procedure was used for every analysis and analyses with applying sample weight, stratum (SDMVSTRA), and primary sampling unit (SDMVPSU) variables [39].

Chi-square test was conducted to test distributions stratified by sociodemographic and behavioral variables for US adults. Spearman correlation analysis was used to examine the correlation between UIC and food group intakes (g/day). We estimated medians and 95% CIs (confidence intervals) of UICs and the prevalence for <50 µg/L UIC between consumers and non-consumers of 12 food groups to assess iodine status according to the consumption of each food group. UIC data are right skewed and thus natural log-transformed to follow a normal distribution when conducting the Spearman correlation and multiple linear regression analyses. Multiple linear regressions were performed to examine the relationship between four significant food groups (dairy products; eggs; breads; beverages) identified from the Spearman correlation analysis as independent variables and UICs after controlling covariates such as survey year, sex, age, race/ethnicity, salt use at the table and in preparation, and iodine-containing supplement use. In the multiple linear regressions, food groups were included as continuous variables per 100 g/day (comparable to ~100 mL of milk/beverage, ~3 slices of bread, or ~2 large eggs) to improve the interpretability [35,38]. Due to missing information on salt use at the table and in preparation, the sample size actually used in the multiple linear regressions was smaller (*n* = 4017) than those for other analyses. Statistical significance was defined as *p* < 0.05.

## 3. Results

The population of this study included 5967 US adults (aged ≥20 years) with 24-h dietary recall, UIC, sociodemographic, and behavioral information. As shown in Table 1, median UIC for the total US adults was 141.4 (95% CI: 133.5–149.2) µg/L. The median UIC for women was lower than the median UIC in men. The lowest median UIC (104.8 (95% CI: 92.2–117.5) µg/L) was found in women who participated in NHANES 2011-2012. Among adults, 7.8% reported taking iodine-containing supplements in the previous 24 h. Similarly, both men (8.2%) and women (7.4%) had low prevalence of using iodine-containing supplements. Over half of US adults (58.1%) added salt occasionally or very often at the table, whereas most US adults (75.5%) never or rarely used salt in cooking or preparing foods in their household. Table salt use was relatively similar between men (58.7%) and women (57.4%). Prevalence of <50 µg/L UIC was different between men and women, with the proportion being higher in women (15.6%) and lower in men (8.5%).

We examined the correlation between food group intake and natural log-transformed UIC in our study sample. We found the UIC was correlated positively with the intake of dairy products, eggs, and breads, and inversely with the intake of beverages (all, *p* < 0.05) (Table 2). Dairy product consumption had the largest positive correlation coefficient with UIC (*r* = 0.15, *p* < 0.01) compared to other food groups. The adjusted means of each food group intake and median UICs and prevalence of <50 µg/L UIC according to the consumption of each food group was described in Table S1. Among the total population, the overall median UIC was 141.4 µg/L. Median UIC and the proportion of <50 µg/L UIC differed by the consumption of dairy products and eggs. Compared to non-consumers of other food group intakes, dairy product non-consumers are more likely to be at risk for iodine deficiency. Median UIC was lowest in dairy product non-consumer group with 117.2 µg/L compared with 149.8 µg/L among dairy product consumers. The prevalence of <50 µg/L UIC was the highest in this group with 14.9%.

Based on multiple linear regressions, after controlling all other general and iodine intake-related characteristics in the model, we found food group intakes were significantly associated with UIC among the total population, men, and women (Table 3). In the total population, when there was a 100 g/day increase in dairy products, eggs, and breads, there was also an increase in UIC of 4%, 5%, and 3%, respectively (all, *p* < 0.05). Additionally, when there was a 100 g/day increase in beverage intake, there was a decrease in UIC of 1% (*p* < 0.001). Among US adults, iodine-containing supplement use in men (*p* < 0.05) and women (*p* < 0.01) was significantly associated with an increasing UIC. In men, for every 100 g/day increase in consumption of dairy products and eggs, there was an increase in UIC of 3% and 6%, respectively (all, *p* < 0.05). In addition, for every 100 g/day increase in beverages, there was a decrease in UIC of 1% (*p* < 0.01). In women, when there was a 100 g/day increase in dairy and beverage intake, there was an increase in UIC of 5% and a decrease of 1%, respectively (all, *p* < 0.01).

## 4. Discussion

In the present study, we investigated the main contributor food groups to US adults’ iodine status using a nationally-representative sample of US adults in the NHANES 2007–2012. In US adults, consumption of dairy products, eggs, and breads was associated with an increasing UIC, whereas beverage consumption was inversely associated with UIC, with some variations between men and women. We found that the consumption of those three food groups positively correlated with UIC is an important contributor to adequate iodine intake compared to the consumption of other food groups, even after controlling for covariates such as survey period, sex, age, race/ethnicity, salt use at the table and in food preparation, and iodine-containing supplement use. When stratified by sex, the associations of egg and bread consumption with UIC were slightly attenuated in women. In addition, iodine-containing supplement use in women contributed more than other factors to variance in UIC.

Previous studies on food sources of iodine identified seafood, dairy products, breads, and eggs as associated with dietary iodine intakes [23,49,50]. It is certainly worthy of note that seafood, including marine fish, shellfish, and seaweed, is well-known as a main sources of dietary iodine [25]. A recently published review by Fuge and Johnson [51] also reported on the importance of the marine environment as dietary sources of iodine in humans in perspective to diet and environmental geochemistry. They stated that populations consuming seafood as a main component in their diet are more likely to have adequate iodine intake because the ocean may play a role as one of the largest iodine-storage mechanisms in the iodine cycle in nature [51]. Particularly, seaweeds living in the ocean can be important concentrators of iodine [52]. However, against this evidence and our expectations, no associations between UIC and fish/seaweed consumption are observed in the present study. These results may suggest that the amount of seafood consumption by Americans may not be sufficient enough to affect UIC significantly [50].

In our study, dairy product consumption had the largest positive correlation with UIC (*r* = 0.2, *p* < 0.01) among the various food groups. Moreover, dairy product consumers have a lower risk for <50 µg/L UIC and adequate iodine intake than non-consumers. The Total Diet Study (TDS) by the Food and Drug Administration (FDA) reported that more than 50% of iodine intake among Americans aged 14 years and older is from dairy products in 2008 [22]. Perrine *et al.* [28] reported that the UIC of pregnant women tends to reflect iodine-sufficient levels as dairy products were consumed frequently. Another major food source of dietary iodine is eggs that have been reported to contribute about 16% of daily iodine requirement (150 µg) in the American diet [27]. In the UK, egg consumption was associated with increasing UIC in school-aged children and childbearing-aged women [53,54]. However, another study with UK adolescents reported no association between UIC and egg consumption [14]. Even though more studies have been conducted in the UK than in the US in recent years, these previous studies address the controversial issues of the effect of egg consumption on UIC, which needs to be investigated further. In contrast, beverage consumption was inversely correlated with UIC in our study. One possible explanation that needs further investigation is goitrogenic chemicals in beverage products. Goitrogens, such as bromine in soft drinks and perchlorate in drinking water, may play a role in iodine uptake by thyroid [2,55,56].

Though the US has few nutritional problems due to iodine deficiency, it is necessary to collect data for identifying iodine intake on a national basis in order to prepare for re-emerging iodine deficiency problems that industrialized countries are beginning to face. The research findings show that food groups such as dairy products, eggs, breads, and beverages play important roles in combating populations’ iodine deficiency. However, some of them contain an issue of unintentional sources of iodine. As previously mentioned, positive correlation between breads and UIC could be due to an iodate (HIO_3_)-containing conditioner, which is added for improving the quality of breads in the manufacturing process [49,57]. While most bread companies provide no information on the iodine amount added to the bread, only a few of them provide information on the added iodine amount; however, this is found to be inaccurate [23]. The same sort of problem happens to dairy products. Since the amount of iodine in dairy products is not regulated by the FDA, it is difficult to accurately determine the iodine amount in dairy products including milk [23]. So far, there is no regulation or supervision on iodine-sanitizing agents used in the production process of the dairy industry. This will require further study to determine whether the iodine content contained in milk comes mainly from the fortified iodine for cattle feed or the iodophor disinfectants [58]. Considering the fact that dairy products and breads are the primary dietary iodine sources in the US, the consumption of these food groups is associated with a chronic exposure to dietary iodine for US adults. Hence, continual research focusing on more precise analytical techniques to evaluate iodine content in main dietary iodine sources is warranted, as well as investigating the associations of food group consumptions with UIC.

This study has several strengths and limitations. This is a unique study aimed at investigating the important food group contributors to iodine status based on a nationally-representative dataset. Most previous studies examined the association between UIC and one or two food groups rather than the entire range of food groups [23,37,38]. Currently, dietary iodine intake cannot be estimated due to the lack of information on iodine contents in the USDA food-composition tables. In order to overcome this challenge, we attempted to determine the associations between food group intakes measured by one 24-h dietary recall and iodine status assessed by UIC. We recognize the limitation of single 24-h dietary recall in estimating habitual dietary intake at an individual level [34] and a single spot UIC for an individual’s iodine status [59]. However, both the 24-h dietary recall data and the spot urine samples were collected at the same time so we may capture the association between intakes and UIC [23]. However, iodine content of foods may vary considerably within the same food group depending on the origin, season, and cooking processes [60]. In order to obtain information on iodine intake within a continually changing population’s food intake patterns, continued efforts in seeking reliable measures of and monitoring of iodine content in each food group are needed.

## 5. Conclusions

To our best knowledge, this study is the first attempt to investigate the important food group contributions to iodine status using a nationally-representative sample of US adults. Our findings indicate that such food groups as dairy, eggs, and breads were positively associated with UIC. Public health interventions may need to aim at achieving iodine adequacy through understanding the importance of consuming iodine-rich food in addition to the use of iodized salt. Future research is needed to extend these initial findings that the association between food group intakes and UIC differs greatly by sex and age. This can provide insight into understanding the role of sex on the relation between food group intakes and iodine status in US populations.

## Figures and Tables

**Table 1:** Urinary iodine concentration by sociodemographic and lifestyle characteristics, NHANES 2007–2012 ^1^.

General Characteristics	Total		Men		Women	
	*n* (Wt’d % ^2^)	Median UIC (95% CI)	*n* (Wt’d %)	Median UIC (95% CI)	*n* (Wt’d %)	Median UIC (95% CI)
Total	5967 (100.0)	141.4 (133.5–149.2) ^3^	3133 (52.3)	158.0 (149.0–167.1)	2834 (47.7)	126.1 (119.5–132.7)
Survey period						
2007–2008	3444 (56.9)	151.4 (139.5–163.4)	1811 (57.5)	166.9 (156.7–177.2)	1633 (56.2)	134.4 (124.1–144.8)
2009–2010	1356 (21.0)	132.0 (121.1–142.9)	706 (21.3)	139.0 (124.6–153.4)	650 (20.7)	126.2 (117.4–135.1)
2011–2012	1167 (22.1)	126.6 (111.3–141.9)	616 (21.1)	150.6 (129.0–172.1)	551 (23.1)	104.8 (92.2–117.5)
Age, years						
20–39	2311 (44.1)	141.5 (132.9–150.1)	1199 (44.5)	149.4 (139.3–159.5)	1112 (43.7)	134.0 (120.4–147.6)
40–59	1997 (38.0)	133.6 (123.8–143.5)	1028 (38.6)	161.0 (143.7–178.3)	969 (37.3)	117.1 (108.2–126.1)
60 and above	1659 (17.9)	159.9 (145.1–174.6)	906 (16.9)	177.4 (164.5–190.4)	753 (19.0)	136.7 (116.5–157.0)
Race/ethnicity ^4^						
NHW	2373 (63.8)	141.5 (130.9–152.2)	1285 (64.3)	166.9 (155.5–178.3)	1088 (63.3)	122.5 (112.5–132.5)
NHB	1388 (13.2)	127.4 (116.3–138.5)	714 (12.0)	128.8 (116.6–141.1)	674 (14.5)	124.9 (111.8–138.0)
All Hispanics	1798 (16.3)	154.2 (144.9–163.5)	898 (16.8)	164.7 (152.9–176.5)	900 (15.7)	146.0 (135.3–156.7)
Other	408 (6.7)	136.6 (117.2–155.9)	236 (6.9)	142.0 (124.1–159.8)	172 (3.1)	119.6 (94.1–145.1)
Iodine-containing supplement use ^5^						
Yes	442 (7.8)	182.4 (155.9–209.0)	243 (8.2)	200.5 (155.6–245.4)	199 (7.4)	168.7 (137.1–200.3)
No	5525 (92.2)	137.6 (129.6–145.7)	2890 (91.8)	153.9 (143.8–164.0)	2635 (92.6)	122.8 (114.6–131.0)
Table salt use ^6^						
Rarely	1760 (41.9)	138.2 (127.9–148.4)	900 (41.3)	152.2 (135.4–168.9)	860 (42.6)	124.6 (114.7–134.6)
Occasionally/very often	2257 (58.1)	144.1 (131.9–156.3)	1251 (58.7)	156.2 (144.0–168.5)	1006 (57.4)	128.6 (114.5–142.7)
Salt use in preparation ^6^						
Never/rarely	3012 (75.5)	140.3 (131.0–149.5)	1595 (75.0)	153.0 (140.2–165.7)	1417 (76.1)	125.6 (116.8–134.3)
Occasionally/very often	1005 (24.5)	146.6 (128.4–164.7)	556 (25.0)	160.5 (142.0–179.0)	449 (23.9)	132.1 (111.9–152.3)
Prevalence of <50 µg/L UIC						
<50 µg/L	665 (11.9)	33.8 (32.1–35.6)	287 (8.5)	33.5 (30.7–36.4)	378 (15.6)	33.8 (31.9–35.7)
≥50 µg/L	5302 (88.1)	161.9 (153.5–170.2)	2846 (91.5)	171.6 (163.7–179.5)	2456 (84.4)	149.7 (141.3–158.1)

^1^ Data are from the National Health and Nutrition Examination Surveys. All data except for sample size are weighted accounting for the complex study design according to the directions of the National Center for Health Statistics. UIC, urinary iodine concentration; ^2^ Wt’d %: Weighted %; ^3^ Median UICs (95% CIs, confidence intervals); ^4^ NHW, non-Hispanic white; NHB, non-Hispanic black; ^5^ Reported taking iodine-containing supplement yesterday; ^6^ The total *n* for salt use variables was 4017.

**Table 2:** Correlation coefficients between urinary iodine concentration and food group intakes in US adults, NHANES 2007–2012 ^1^.

Food Group (g/Day) ^2^	UIC (µg/L)		
	Total	Men	Women
Dairy products	0.151 **^,3^	0.155 **	0.142 **
Meat/poultry	−0.017	−0.037 **	0.028
Fish/seaweed	0.006	−0.001	0.001
Eggs	0.051 **	0.042 *	0.045 *
Legumes/nuts/seeds	−0.014	−0.033	−0.007
Breads	0.052 **	0.047 *	0.057 **
Other grain products	0.003	−0.025	0.004
Fruits	−0.021	−0.022	−0.033
Vegetables	−0.027	−0.014	−0.038
Sugars/sweets	0.022	0.023	0.017
Fats/oils	0.002	0.016	−0.016
Beverages	−0.147 **	−0.159 **	−0.188 **

^1^ Data are from the National Health and Nutrition Examination Surveys. All data except for sample size are weighted accounting for the complex study design according to the directions of the National Center for Health Statistics. UIC, urinary iodine concentration; ^2^ We created 12 major food groups (g/day) including food groups known to be high in iodine content (dairy products; meat/poultry; fish/seaweed; eggs; legumes/nuts/seeds; breads; other grain products; fruits; vegetables; sugars/sweets; fats/oils; beverages); ^3^ Spearman correlation coefficients were calculated using natural log-transformed data (* *p* < 0.05, ** *p* < 0.01).

**Table 3:** Association of urinary iodine concentrations with food group intakes and sociodemographic and lifestyle characteristics in US adults, NHANES 2007–2012 ^1^.

	Total (*n* = 4017)				Men (*n* = 2151)				Women (*n* = 1866)			
	Estimate	95% CI ^2^		*p*-Value ^3^	Estimate	95% CI		*p*-Value	Estimate	95% CI		*p*-Value
Intercept	5.20	5.05	5.36	<0.001 **	5.15	4.96	5.35	<0.001 **	5.03	4.85	5.22	<0.001 **
Dairy products ^4^, per 100 g/day	0.04	0.02	0.06	<0.001 **	0.03	0.01	0.06	0.006 **	0.05	0.02	0.07	0.002 **
Eggs, per 100 g/day	0.05	0.01	0.10	0.026 *	0.06	0.01	0.12	0.027 **	0.04	−0.05	0.12	0.384
Breads, per 100 g/day	0.03	0.00	0.06	0.045 *	0.01	−0.02	0.05	0.501	0.05	−0.01	0.12	0.119
Beverages, per 100 g/day	−0.01	−0.01	−0.01	<0.001 **	−0.01	−0.01	0.00	<0.001 **	−0.01	−0.02	−0.01	<0.001 **
Survey year												
2007–2008	Reference				Reference				Reference			
2009–2010	−0.13	−0.24	−0.03	0.014 **	−0.18	−0.30	−0.06	0.004 **	−0.08	−0.24	0.08	0.326
2011–2012	−0.11	−0.24	−0.02	0.086 *	−0.03	−0.17	0.12	0.722	−0.19	−0.35	−0.02	0.028 *
Sex												
Men	−0.22	−0.29	−0.14	<0.001 **	‒	‒	‒	‒	‒	‒	‒	‒
Women	Reference				‒	‒	‒	‒	‒	‒	‒	‒
Age, years												
20–39	Reference				Reference				Reference			
40–59	−0.08	−0.17	0.01	0.061	0.05	−0.04	0.14	0.294	−0.22	−0.33	−0.10	0.001 **
60 and above	−0.02	−0.14	0.11	0.793	0.07	−0.06	0.20	0.302	−0.08	−0.25	0.10	0.383
Race/ethnicity ^5^												
NHW	Reference				Reference				Reference			
NHB	−0.09	−0.21	0.03	0.123	−0.17	−0.30	−0.04	0.012 *	−0.01	−0.15	0.13	0.873
All Hispanics	−0.02	−0.11	0.07	0.646	−0.09	−0.21	0.02	0.116	0.07	−0.06	0.20	0.276
Other	−0.01	−0.17	0.16	0.924	0.08	−0.29	0.12	0.422	0.07	−0.13	0.27	0.467
Iodine-containing supplement use ^6^												
Yes	0.31	0.14	0.48	<0.001 **	0.19	0.01	0.36	0.041 *	0.41	0.19	0.62	<0.001 **
No	Reference				Reference				Reference			
Table salt use ^7^												
Rarely	0.02	−0.08	0.12	0.685	0.04	−0.09	0.17	0.550	−0.02	−0.13	0.10	0.786
Occasionally/very often	Reference				Reference				Reference			
Salt use in preparation ^7^												
Never/rarely	Reference				Reference				Reference			
Occasionally/very often	0.04	−0.06	0.14	0.430	0.03	−0.09	0.15	0.631	0.04	−0.12	0.20	0.608

^1^ Data are from the National Health and Nutrition Examination Surveys. All data except for sample size are weighted accounting for the complex study design according to the directions of the National Center for Health Statistics. Food groups (dairy products, eggs, breads, and beverages) correlated significantly with urinary iodine concentration (UIC) were included in multiple linear regressions; ^2^ Estimates (95% Confidence Intervals) are from separate multiple linear regression analyses for total population, men, and women. The dependent variable is the natural log-transformed UIC; ^3^ The change in UIC associated with a 1 g/day change in food group intake is not very meaningful. We expressed intake per 100 g/day of these food groups; ^4^ *p* for trend obtained from multiple linear regression analysis (* *p* < 0.05, ** *p* < 0.01); ^5^ NHW, non-Hispanic white; NHB, non-Hispanic black; ^6^ Reported taking iodine-containing supplement yesterday; ^7^ The total *n* for salt use variables was 4017.

## Supplementary Materials

The following are available online at http://www.mdpi.com/2072-6643/8/6/325/s1. Table S1: Median urinary iodine concentrations (UICs) and prevalence of <50 µg/L UIC in relation to each food group consumption in US adults, NHANES 2007–2012.
