# Supplementary material for: Food Group Intakes as Determinants of Iodine Status among US Adult Population
Source: Nutrients. 2016 May 26;8(6):325. doi: 10.3390/nu8060325 (PMC4924166; doi:10.3390/nu8060325)
Supplement: Supplementary file 1 [file nutrients-08-00325-s001.docx]

Supplementary Materials: Food Group Intakes as Determinants of Iodine Status among US Adult Population

Kyung Won Lee, Dayeon Shin, Mi Sook Cho and Won O. Song

**Table S1.** Median urinary iodine concentrations (UICs) and prevalence of <50 µg/L UIC in relation to each food group consumption in US adults, NHANES 2007–2012 ^1^.

| **Food Group** | | **Subjects, *n*** | **Intake (g/Day)** | | **UIC** | | | | **Prevalence of <50 µg/L UIC** | | |
| --- | --- | --- | --- | --- | --- | --- | --- | --- | --- | --- | --- |
|  |  |  | **Mean** | **SE** | **Median** | **95% CI** ^2^ | | ***p*-Value** ^3^ | **%** | **SE** ^4^ | ***p*-Value** ^5^ |
| Dairy products ^6^ | NC ^7^ | 1341 | 0 |  | 117.2 | 111.5 | 122.9 | <0.001 ** | 14.9 | 1.6 | <0.01 ** |
|  | C | 4626 | 261.7 | 8.4 | 149.8 | 139.7 | 160.0 |  | 11.7 | 0.7 |  |
| Meat/poultry | NC | 978 | 0 |  | 135.1 | 114.7 | 155.4 | 0.658 | 14.0 | 1.5 | 0.249 |
|  | C | 4989 | 232.8 | 3.9 | 140.5 | 132.7 | 148.3 |  | 12.0 | 0.8 |  |
| Fish/seaweed | NC | 4792 | 0 |  | 140.6 | 133.0 | 148.3 | 0.615 | 12.2 | 0.7 | 0.603 |
|  | C | 1175 | 157.9 | 5.8 | 135.1 | 117.5 | 152.6 |  | 13.0 | 1.4 |  |
| Eggs | NC | 4546 | 0 |  | 134.1 | 126.9 | 141.2 | <0.001 ** | 12.8 | 0.8 | 0.031 * |
|  | C | 1421 | 118.8 | 3.2 | 166.8 | 151.6 | 181.9 |  | 10.6 | 1.0 |  |
| Legumes/nuts/seeds | NC | 4192 | 0 |  | 141.2 | 131.5 | 150.8 | 0.529 | 11.6 | 0.8 | 0.189 |
|  | C | 1775 | 102.9 | 3.1 | 136.6 | 125.1 | 148.2 |  | 14.0 | 1.5 |  |
| Breads | NC | 682 | 0 |  | 131.5 | 116.5 | 146.5 | 0.340 | 12.8 | 1.8 | 0.805 |
|  | C | 5285 | 134.0 | 2.1 | 141.0 | 132.9 | 149.1 |  | 12.3 | 0.8 |  |
| Other grain products | NC | 2173 | 0 |  | 136.4 | 124.3 | 148.5 | 0.518 | 13.6 | 1.3 | 0.182 |
|  | C | 3794 | 288.6 | 4.9 | 141.4 | 131.7 | 151.0 |  | 11.6 | 0.8 |  |
| Fruits | NC | 2778 | 0 |  | 141.4 | 129.4 | 153.4 | 0.675 | 11.4 | 1.1 | 0.237 |
|  | C | 3189 | 297.2 | 6.8 | 138.9 | 128.9 | 148.9 |  | 13.3 | 1.0 |  |
| Vegetables | NC | 1195 | 0 |  | 138.6 | 121.6 | 155.7 | 0.970 | 13.8 | 1.4 | 0.245 |
|  | C | 4772 | 207.1 | 4.9 | 139.5 | 129.9 | 149.1 |  | 12.0 | 0.8 |  |
| Sugars/sweets | NC | 2632 | 0 |  | 139.4 | 129.4 | 149.3 | 0.997 | 11.8 | 0.8 | 0.334 |
|  | C | 3335 | 37.1 | 1.8 | 139.2 | 129.5 | 149.0 |  | 12.9 | 1.0 |  |
| Fats/oils | NC | 3487 | 0 |  | 143.4 | 132.5 | 154.4 | 0.313 | 11.2 | 0.8 | 0.078 |
|  | C | 2480 | 23.9 | 0.7 | 135.6 | 125.2 | 146.1 |  | 13.8 | 1.2 |  |
| Beverages | NC | 34 | 0 |  | 236.0 | 163.6 | 308.4 | 0.116 | 4.0 | 3.0 | 0.118 |
|  | C | 5933 | 2305.1 | 45.1 | 139.1 | 130.8 | 147.4 |  | 12.4 | 0.7 |  |

^1^ Data are from the National Health and Nutrition Examination Surveys. All data, except for sample size, are weighted accounting for the complex study design according to the directions of the National Center for Health Statistics. ^2^ Data values are reported as medians (95% CIs, confidence intervals). ^3^ *p*-values for comparison of median UIC by food group intakes are from the chi-square test comparing whether the proportion above or below the overall median UIC differs by these groups. ^4^ Weighted percentage (SE, standard error). ^5^ Chi-square test for the prevalence of <50 µg/L UIC between non-consumers and consumers of each food group (* *p* < 0.05, ***p* < 0.01). ^6^ We created 12 major food groups, including food groups known to be high in iodine content (dairy products; meat/poultry; fish/seaweed; eggs; legumes/nuts/seeds; breads; other grain products; fruits; vegetables; sugars/sweets; fats/oils; beverages). ^7^ NC, non-consumers; C, Consumers.
